# Ectopic thymoma presenting as a giant intrathoracic mass: A Case Report

**DOI:** 10.1186/1749-8090-7-68

**Published:** 2012-07-16

**Authors:** Tomoyoshi Takenaka, Teruyoshi Ishida, Yoshinori Handa, Shinichi Tsutsui, Hiroyuki Matsuda

**Affiliations:** 1 Department of Surgery, Hiroshima Red Cross Hospital and Atomic Bomb Survivors Hospital, 1-9-6 Senda-Machi, Naka-ku, Hiroshima, Japan

**Keywords:** Giant thymoma, Intrathoracic tumor, Ectopic thymoma

## Abstract

Thymoma is an epithelial neoplasm of the thymus, which commonly lies in the anterior mediastinum and, therefore, an intrathoracic origin is considered to be rare. This report presents a case of giant thymoma arising in the thoracic cavity. A 61-year-old male presented with a chronic cough and breathlessness. Chest CT revealed a well enhanced giant mass approximately 18 cm in diameter in the right thoracic cavity. FDG-PET showed that the SUVmax of the tumor was 5.0 in the center and almost 2.5 in the surrounding area. A trans- bronchial needle biopsy was performed to find atypical cells. Surgery was scheduled based on the radiological and histological findings. A well-defined giant mass in the thoracic cavity, measuring 18 × 14.5 × 11 cm had undergone expansive growth without apparent invasion. The tumor was completely resected without combined resection of the other organs. The weight of the tumor was 1350 g. The tumor was histologically diagnosed to be type AB thymoma according to the World Health Organization classification and Masaoka stage IIB.

## Background

Thymoma is an epithelial neoplasm of the thymus, which commonly lies in the anterior mediastinum and ectopic thymomas account for only 4% of all thymomas [1]. Most ectopic thymomas present as superior or inferior mediastinum tumors, therefore intrathoracic origin was rare [1-3]. Although surgery is the most effective treatment modality for thymomas, in some cases, it is difficult to excise due to involvement of surrounding organs or tumor size. This report presents a case of ectopic thymoma that presented as a giant intrathoracic tumor histologically diagnosed as type AB thymoma and Masaoka stage IIB.

## Case presentation

**Figure 1:**
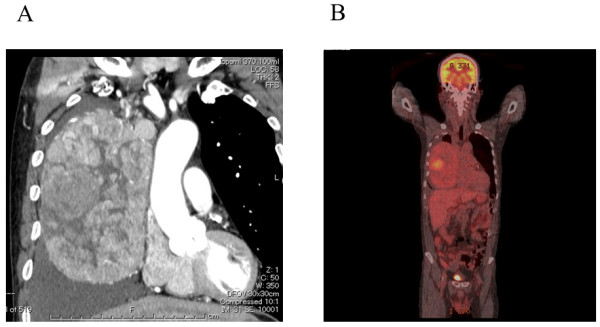
**(A) Chest CT showing a huge mass, measuring 18 × 14 × 11 cm in diameter in the right thoracic cavity.(B)** FDG-PET revealed that SUVmax of the tumor was 5.0 in the center and almost 2.5 in the surrounding areas.

**Figure 2:**
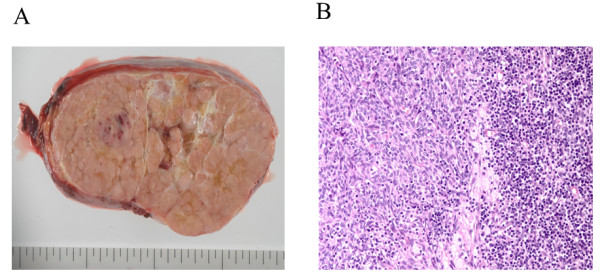
**(A) Gross pathologic finding of the tumor: an encapsulated elastic mass.** The cut surface of tumor revealed a light brown color, an internal lobulated structure with a portion of capsulation invasion. **(B)**Histological findings of the tumor: the tumor shows a proliferation of spindle-shaped epithelial cells with many lymphocytes (original magnification: 100×).

A 61-year-old male presented with chronic cough and breathlessness. Chest CT revealed a well enhanced giant mass, approximately 18 cm in diameter in the right thoracic cavity (Figure 1). FDG-PET showed that the SUVmax of the tumor was 5.0 in the center and almost 2.5 in the surrounding areas (Figure 1). A trans-bronchial biopsy was performed and atypical cells were detected from the giant tumor. An intrathoracic malignant tumor was suspected, therefore surgical resection was performed. The CT findings suggested that the mass had not invaded surrounding organs, but had some adhesion to the right lung and superior vena cava. Surgical access to the mass was accomplished through a median sternotomy with a 4th intercostal thoracotomy of the right chest wall. A well-defined giant mass measuring 18 × 14.5 × 11 cm was found in the right thoracic cavity. The feeding vessels of the tumor were branching from internal thoracic vessels and collateral blood vessels were extensive surrounding the giant tumor. The tumor was completely resected without combined resection of the other organs. The weight of the tumor was 1350 g. The cut surface of tumor revealed a light brown color, an internal lobulated structure with a portion of capsulation invasion (Figure 2). A microscopic examination showed the tumor to contain spindle shaped cells with a lymphocyte rich component (Figure 2). Immunohistochemistry showed predominantly lymphocytes expressing CD1a, TdT and CD5. The spindle cells of the tumor were all negative for EMA, cytokeratin, CAM5.2,αSMA, S100, CD5, TTF-1 and CD34. These histopathological findings indicated that the tumor was a type AB thymoma according to the World Health Organization classification and Masaoka stage IIB. The postoperative course was uneventful, and there has been no evidence of recurrence 6 months after the surgery.

## Discussion

Thymomas develop mainly in the thymus and are usually located in the anterior mediastinum, only 4% of the tumors are ectopic tumors [1]. Ectopic thymomas have been reported in the neck, middle or posterior mediastinum, lung and pleura, a few reports have described giant intrathoracic tumors [2,3].

FDG-PET showing increased uptake of FDG is suggestive of malignant potential and SUVmax correlates with malignancy in mesenchymal tumors. Several authors reported a significant correlation between the histological subtype of thymicepitherial tumors and FDG-PET accumulation [4,5]. Who type A, AB and B1 tumors have significantly lower SUV than that of the other types of tumors [4,5]. In addition, SUVmax has a close correlation with the Masaoka stages [5]. SUVmax of the tumor in the current case was not very high in the proportion to the size. Fortunately, the tumor did not invade the heart or great vessels, therefore curative resection could be performed. The tumor was diagnosed to be type AB thymoma according to the World Health Organization classification and Masaoka stage IIB.

Although thymomas can present as huge masses, the symptoms and stage may not always correlate with tumor size. A large tumor size is a significantly poor prognostic factor of thymomas [6]. Limmer et al. reviewed previously reported giant thymomas [7]. According to the article, all of the giant tumors were type A, AB and B1 according to WHO classification [7]. Interestingly, although a large tumor size is a poor prognostic factor, the resected giant thymomas tended to be low-grade thymomas.

## Conclusion

This report presented a very rare case of an intrathoracic giant thymoma, histologically diagnosed as type AB Masaoka stage IIB. Surgical resection must be considered regardless of tumor size if curative resection is possible. FDG-PET is useful modality for preoperatively evaluating the grade of thymomas.

## Consent

Our Institution and the patient provided Clinical Consent for the publication of this case.

## Competing interest

All the authors declare that they have not competing interest.

## Author’s contribution

TT: concept and design, writing the article. YH, ST, HM made a critical review of the manuscript. TI: final approval of the article. All authors read and approved the final manuscript.
